# Visual setup of logical models of signaling and regulatory networks with ProMoT

**DOI:** 10.1186/1471-2105-7-506

**Published:** 2006-11-17

**Authors:** Julio Saez-Rodriguez, Sebastian Mirschel, Rebecca Hemenway, Steffen Klamt, Ernst Dieter Gilles, Martin Ginkel

**Affiliations:** 1 Max-Planck-Institute for Dynamics of Complex Technical Systems, Sandtorstr. 1, 39106 Magdeburg, Germany

## Abstract

**Background:**

The analysis of biochemical networks using a logical (Boolean) description is an important approach in Systems Biology. Recently, new methods have been proposed to analyze large signaling and regulatory networks using this formalism. Even though there is a large number of tools to set up models describing biological networks using a biochemical (kinetic) formalism, however, they do not support logical models.

**Results:**

Herein we present a flexible framework for setting up large logical models in a visual manner with the software tool ProMoT. An easily extendible library, ProMoT's inherent modularity and object-oriented concept as well as adaptive visualization techniques provide a versatile environment. Both the graphical and the textual description of the logical model can be exported to different formats.

**Conclusion:**

New features of ProMoT facilitate an efficient set-up of large Boolean models of biochemical interaction networks. The modeling environment is flexible; it can easily be adapted to specific requirements, and new extensions can be introduced. ProMoT is freely available from .

## Background

The analysis of regulatory mechanisms using Boolean formalisms is an important technique [1], and has been successfully applied to systems of moderate size, e.g. [2-4]. Furthermore, a tool (GINSim) has been developed to set up and analyze logical networks [5].

Recently, new techniques based on a logical formalism – in combination with graph-theoretical methods applied to the underlying interaction graph – have been proposed for the analysis of large-scale signaling and regulatory networks [6]. These methods have been implemented in *CellNetAnalyzer *(*CNA*), allowing structural analysis of large networks within a GUI [7].

In *CNA*, the user should provide a graphical map of the network, a mathematical (textual) input of the network structure, and a mapping from the latter to the earlier. However, the procedure for setting up large-scale networks by hand, of both the graph and text, can be a cumbersome and error-prone task. There are many tools available to set up models describing signaling networks as a biochemical reaction network, such as CellDesigner, JDesigner, and ProMoT [8-10]. However, to the best of our knowledge, there is currently no tool available that allows the visual setup of large logical networks, and has the ability to export both the mathematical model and the graphical representation together. Note that GINSim allows to define visually the elements of a network and their connections, but the detailed logical information must be included via textual rules.

Therefore, we have extended the abilities of ProMoT [10] to fill this gap. First, we setup a library of basic logical elements (compound, and, not, etc.), which also possess properties that contain additional information. Subsequently, we developed a visualization component that allows the user to customize the representation of the logical model. Finally, we created exports in several formats which provide an input for analysis packages. In the following, we describe briefly these developments and show their applicability via a toy model, as well as using a realistic large model of T-cell signaling recently published [11].

## Implementation

**Figure 1:**
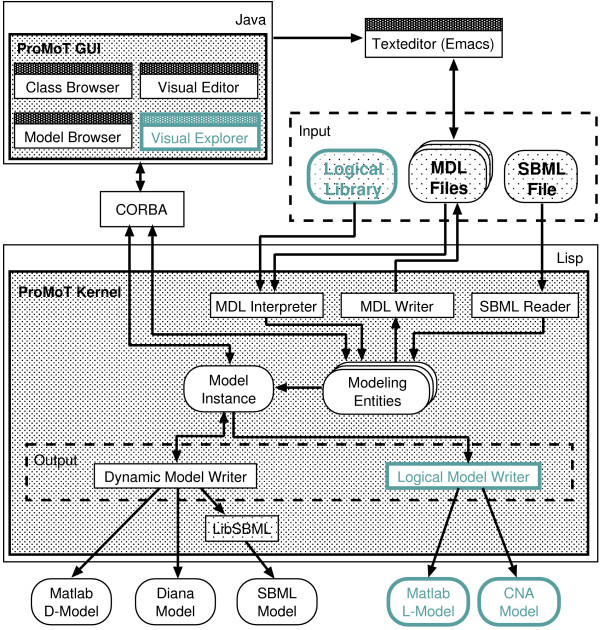
**Architecture of ProMoT**. Diagram of the software architecture of ProMoT. Added or extended software components are highlighted in green and bold face. Models can be set up either via the GUI or a text editor using the MDL format. The kernel can read and write model representations to files in MDL format. It can also read SBML models for Systems Biology. Internally models are represented as classes with inheritance and aggregation mechanisms. The user can manipulate and visualize these classes via the GUI. For analysis, a model instance is created and processed by one of the different writers for generating the input of an analysis system.

All aspects of this work are implemented in ProMoT [10]. ProMoT itself consists of two main units, the kernel (written in Lisp) and the GUI (written in Java), which are interfaced via a CORBA middleware (see Figure 1). The kernel contains the memory-representation of the models and can read and write storage formats. The GUI is designed for visually constructing models (via the *Visual Editor*) and for the interactive exploration and further visual alteration of an existing model (via the *Visual Explorer*). Additionally, models can also be setup via ProMoT's modeling language MDL (Model Definition Language). The modeling elements are organized in a modeling library and can be re-used. ProMoT supports the development of modules as classes in an object-oriented inheritance hierarchy. ProMoT has been used so far for developing structured dynamic models based on differential-algebraic equations in the fields of System Biology and Process Engineering. These models can be simulated in Matlab or our own simulation environment DIANA. Additionally for biochemical models an import and export option for the Systems Biology Markup Language (SBML) is implemented. For the logical networks, a new type of models has been added to ProMoT. The model definition and handling works in the same way as for the dynamic models, only the respective modeling libraries and the output system are specific for logical interaction hypergraphs as defined in [6].

The structure of the ProMoT implementation with the multiple output formats allows to use the features of different analysis and simulation tools without the need of reimplementing the models. Also the graphical representation and editing can be applied in almost the same way for the different kinds of models, which makes it easier for the modelers.

The *Visual Explorer *is a new feature of ProMoT that provides a versatile visualization. It uses several ZUI (Zoomable User Interface) functionalities of the Piccolo toolkit [12]. Thus, highly interactive and adaptive visualization can be created to represent large and complex logical networks and facilitate their convenient exploration. The visualization is altered applying the concept of visual scenarios (for more details see section Visualization). A tutorial with a detailed explanation on how to install ProMoT accompanies this paper (see Additional File 1), as well as ProMoT'source code (see Additional File 2).

## Results

### Definition of a library of basic elements

**Figure 2:**
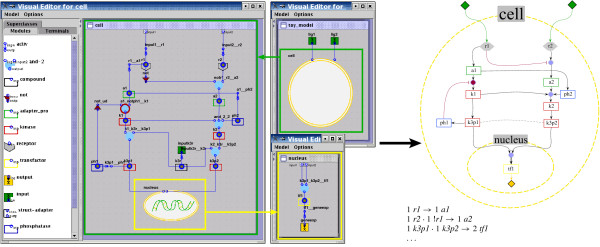
**Screenshot of the Visual Editor of a toy model in ProMoT (left) and of its visually processed export (right), (e.g. to CellNetAnalyzer)**. The text in the bottom of the right figure shows an incomplete textual export (where '!' denotes Not, '·' represents AND, and '→' activation. Note how, in the *Visual Editor*, inhibition is encoded by a not element between the compound and the gate and, after applying the visual scenario, it is represented by a single red-colored connection line (but could be easily changed to another color value). Additionally, the direction is indicated by an arrow symbol (different for activation and inhibition), which is implicitly defined in the mathematical description. Furthermore, the element k3r has been hidden, since it belongs to the class reservoir and has thus no biological meaning. Complete textual exports, as well as alternative visualizations, can be found in Tables 1-2 and Figure 4, respectively.

There are two main classes in the modeling library, compound (representing an state), and gate (defining a logical interaction between compounds). Applying ProMoT's object-oriented modeling paradigm [10], subclasses of a certain class can be easily defined. For example, we have defined subclasses of the class compound (e.g. receptor, kinase, adapter, and reservoir), which are all mathematically equivalent to compound but can be specifically considered in the later visualization process (see Figure 2).

To define different logical connections among the elements we subclassed the class gate into activ (to describe a causal one-to-one relation between two compounds), and (to define the requirement of several elements to active a certain compound), and not (to express a negative effect, i.e., an inhibition). AR Or gate can be implemented by including several activ elements pointing at a certain compound. Since any logical connection can be described as a combination of ANDS, ORS, and NOTS[6], the set of basic elements described above allows to set up any logical network of arbitrary size. In addition, to describe cases where the logic is unclear [6], we have also included the class somehow which represents logical gates with partially incomplete truth tables.

Finally, the classes input and output allow to define the incoming and outgoing signals of the model, respectively.

**Figure 3:**
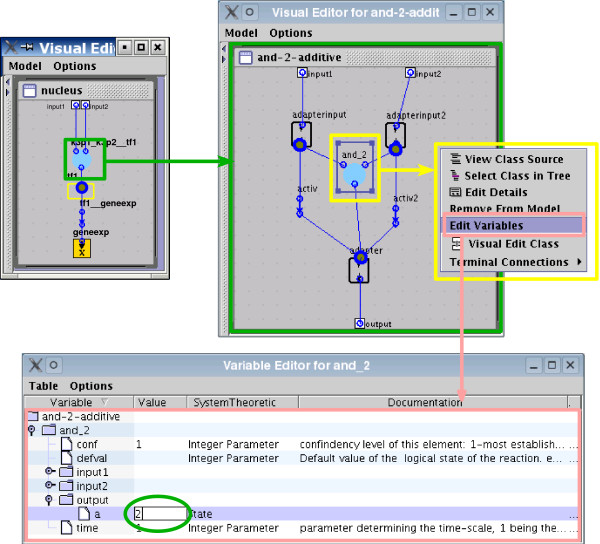
**Illustration of the setting of parameters by the encoding of multiple levels**. Properties like the *multilevel *variable can be edited in the variable editor. The parameter a (level of activation) default equals 1, and can be modified to encode multilevel logical operations. For example, in the toy model (see Figure 2), tf1 reaches a level 2 if both k3p1 and k3p2 are active. This is set up in ProMoT via a gate of the class AND where the output has a = 2).

Properties can be easily added to the different classes. For example, we have defined parameters for the default value and time-scale [6], which are exported with the model. Multiple levels (i.e., discretizing the states into more than two (0,1) levels) is also implemented using the properties of the gates. Inputs and outputs of all gates posses a parameter (with default value 1) encoding the level: the parameter of the input defines which state must reach the start node to activate the target, and the parameter of the output the level the target will reach (see Figure 3). Additionally, all elements have a documentation, which can also be exported with the model.

### Exploiting the modularity

ProMoT allows to set up models in a modular manner [10]. This unique property can be used to define different submodules, either physically delimited (e.g. the nucleus, see Figure 2) or comprising a particular submodel (e.g. the machinery of the cell cycle regulation). The modules can be reused. For example, a module of the ubiquitous MAPK Cascade may appear in several models or several times in one model. So, it is only required to set up this module once and then use it several times, facilitating and speeding up the building procedure.

### Visualization

The elements described above enables a precise setup of a mathematical model, but the representation is certainly not biologically intuitive. Since the models are characterized by many components and interactions, visualization aspects are of great importance [13]. We tackle this issue with scenario-based visualization techniques (compare Figure 2 left and right) implemented in the *Visual Explorer*, a new component to the GUI of ProMoT (see Figure 1 and Section Implementation).

A visual scenario describes a set of mapping functions that define the visual properties of elements (shape, size, color, etc.). In this manner the visualization of the entire network can be adapted towards a more biological meaning and/or user's preferences. The biological intuitiveness is realized by (1) aggregation of all modules and hierarchies in a single visualization, (2) hiding of elements that are necessary for the mathematical description but without a clear biological interpretation, and (3) altering the visual properties of certain elements to achieve a biologically more intuitive representation.

The following examples illustrate the process of designing a biological motivated representation of the model, and the resulting map is depicted in Figure 2 (right side). Elements of the class not are hidden, and the corresponding information (that a certain influence has a negative effect) is coded in the color of the line. Another class specifically treated is the reservoir. Elements of this class are used when several pools of a molecule are considered. This is a common situation, for example, if two subsets of a certain molecule with distinct properties, typically a different localization in the cell or different regulation, are to be modeled. For example, in the toy model there are two pools for the kinase k3, k3p1 and k3p2, which are regulated differently. In this case, a 'reservoir' is included which is required for the activation of both pools. This allows simultaneous knock-outs of both pools, but this is also a mathematical entity without a clear biological interpretation. Hence, elements of the class reservoir are automatically hidden, and the line connecting them to the corresponding pools is dashed. In this way, the mathematical information (there is a reservoir present) is maintained in the graphical representation, but coded in such a way that it is not confused with other kind of information.

**Figure 4:**
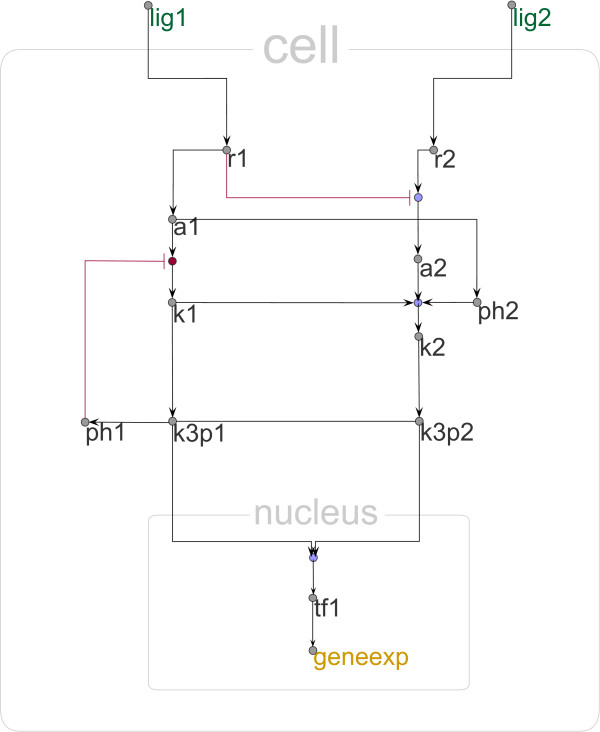
**Alternative visualizations**. Using the concept of visual scenarios the whole network can be visually altered. Here, a new visual scenario towards a more abstract representation is defined as an alternative to the scenario used in Figure 2. All elements are reduced in size (the label is now positioned outside), proteins have a uniform color, connection lines are orthogonalized, and the border of cell and nucleus are de-emphazised to draw the attention to both logical operations and connections.

The visual scenario including the visual properties of the elements can be easily edited using a setup dialog. For example, an alternative representation, matching different preferences, is depicted in Figure 4.

### Generation of models for analysis

**Figure 5:**
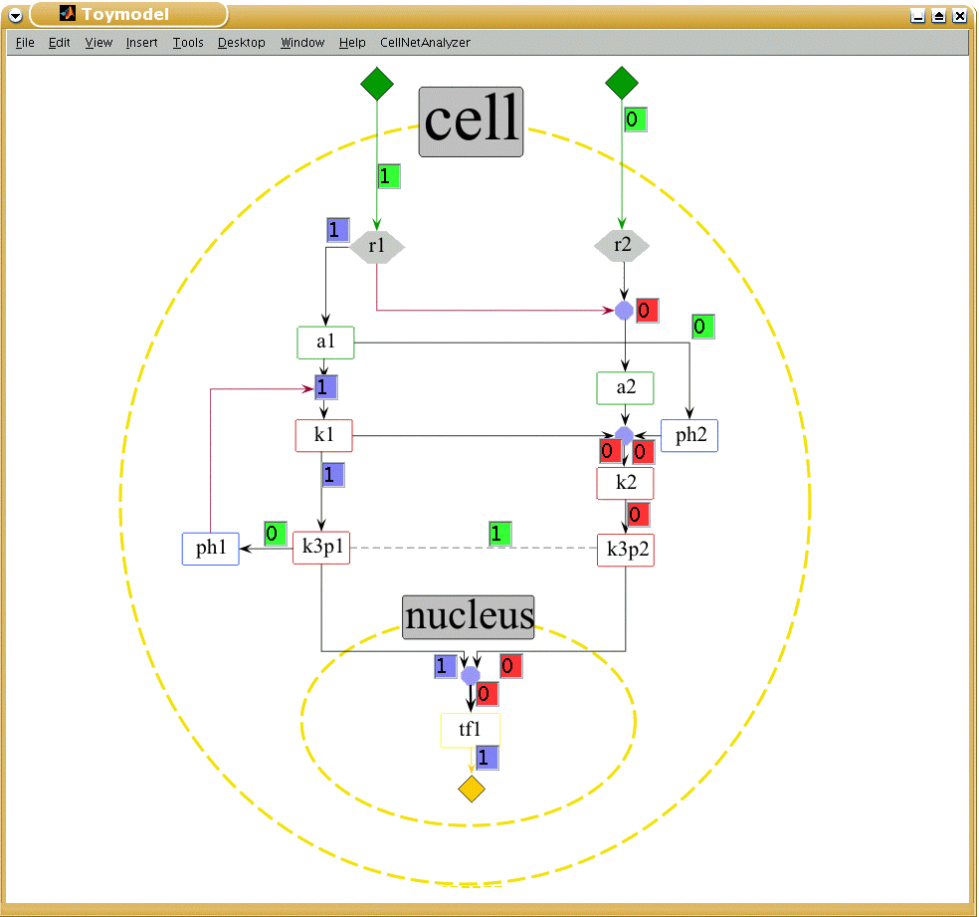
**Screenshot of the toy model exported to CellNetAnalyzer**. Both the textual description (see Table 1) and the map of the toy model of Figure 2 were exported and loaded into *CNA*. Note that the position of the text boxes (x, y) were automatically generated by ProMoT. The figure shows an analysis where the receptor R1 was activated, R2 was not, and the element with a time scale 2 [6] where considered to be inactive. Small text boxes display the signal flows along the hyperarcs (green boxes: fixed values prior to computation; blue boxes: hyperarcs activating a species (signal flow is 1); red boxes: hyperarcs which are not active (signal flow is 0)).

As mentioned before, ProMoT does not perform the analysis of the models, but instead generates input for analysis packages like *CellNetAnalyzer *(CNA), see Figure 5. In this process the model classes, generated by the user, get instantiated. Since analysis packages usually don't consider modularity and structure, only the flat network of leafnode elements in the model, defined in the modeling library, are extracted. Even though for the model setup the user can construct complex classes in a hierarchical manner, for the export only a network based on the basic elements that the analysis tools can handle (compounds and gates) can be exported. These leafnode elements and their connections are translated to the input format of the analysis software, based on their mathematical semantics and specific parameters given by the user. Additionally, in the case of tools which support a graphical interface, graphics and layout informations can also be generated.

Even though there has recently been an initiative to define a XML format for boolean networks – GinML, see [5], – there is currently no established standard for logical networks: SBML only supports kinetic models. Therefore, we have first implemented the generation of input for *CNA *(see Table 1 and 2) and some other simple formats such as a transition function for the whole boolean network expressed as MATLAB code (see Table 2). Other exports (e.g. for GinML) can be easily added. However, we think that first a well-defined standard for discrete and logical models should be established by finding some common denominator of different tools for discrete analysis of cellular network (preferably as part of the SBML initiative), to facilitate model exchange.

**Table 1:** Export to CellNetAnalyzer

Name	Description		Def	Sh	t	-	x y	- - -
r1	1 a1 + 1 !ph1 = 1 k1	|	#	1	1	0	310 346	1 1 0
r2	1 a1 = 1 ph2	|	#	0	2	0	689 285	1 1 0
r3	= 1 r1	|	0	0	1	0	373 134	1 1 0
r4	= 1 r2	|	0	0	1	0	621 77	1 1 0
r5	1 inputk3r = 1 k3r	|	1	0	1	0	485 493	1 1 0
r6	1 k1 + 1 k3r = 1 k3p1	|	#	0	1	0	317 434	1 1 0
r7	1 k2 + 1 k3r = 1 k3p2	|	#	0	1	0	624 474	1 1 0
r8	1 k3p1 = 1 ph1	|	#	0	2	0	259 493	1 1 0
r9	1 !r1 + 1 r2 = 1 a2	|	#	0	1	0	633 269	1 1 0
r10	1 r1 = 1 a1	|	#	0	1	0	322 188	1 1 0
r11	1 a2 + 1 k1 = 1 k2	|	#	0	1	0	596 410	1 1 0
r12	1 a2 + 1 ph2 = 1 k2	|	#	0	1	0	629 411	1 1 0
r13	1 tf1 =	|	#	0	1	0	472 718	1 1 0
r14	1 k3p1 = 1 tf1	|	#	0	1	0	429 626	1 1 0
r15	1 k3p2 = 1 tf1	|	#	0	1	0	496 625	1 1 0
r16	1 k3p1 + 1 k3p2 = 2 tf1	|	#	0	1	0	471 653	1 1 0

The table shows the file exported to *CNA *[6], where Def means default value, # codes an non-determined value, Sh whether an element posses an incomplete truth table (1) (i.e., is a somehow, see main text) or not (0), t the time scale, and x and y the positions in the map. In *CNA *an AND connection is coded via '+' instead of '·' and the exclamation mark '!' indicates a NOT.

**Table 2:** Matlab function expressing the boolean network.

function new = rules toy model(setnow, lnow, nodes)		
new = zeros(lnow,1);		
Lig1 = 1;	Lig2 = 2;	A1 = 3;
A2 = 4;	Inputk3r = 5;	K1 = 6;
K2 = 7;	K3p1 = 8;	K3p2 = 9;
K3r = 10;	Ph1 = 11;	Ph2 = 12;
R1 = 13;	R2 = 14;	Geneexp = 15;
Tf1 = 16;		
		
for i = 1:lnow		
inode = setnow(i) ;		
switch inode		
	case A1	new(i) = nodes(R1);
	case A2	new(i) = and((nodes(R1) == 0), nodes(R2));
	case K1	new(i) = and((nodes(Ph1) == 0), nodes(A1));
	case K2	new(i) = or(and(nodes(A2), nodes(Ph2)), and(nodes(K1), nodes(A2)));
	case K3p1	new(i) = and(nodes(K1), nodes(K3r));
	case K3p2	new(i) = and(nodes(K3r), nodes(K2));
	case K3r	new(i) = nodes(Inputk3r);
	case Ph1	new(i) = nodes(K3p1);
	case Ph2	new(i) = nodes(A1);
	case R1	new(i) = nodes(Lig1);
	case R2	new(i) = nodes(Lig2);
	case Geneexp	new(i) = nodes(Tf1);
	case Tf1	new(i) = or(or(and(nodes(K3p1), nodes(K3p2)), nodes(K3p2)), nodes(K3p1));
end		
end		

Alternative export of the toy model for simulations in Matlab, e.g. as used in [2].

### Proof of principle for large networks

**Figure 6:**
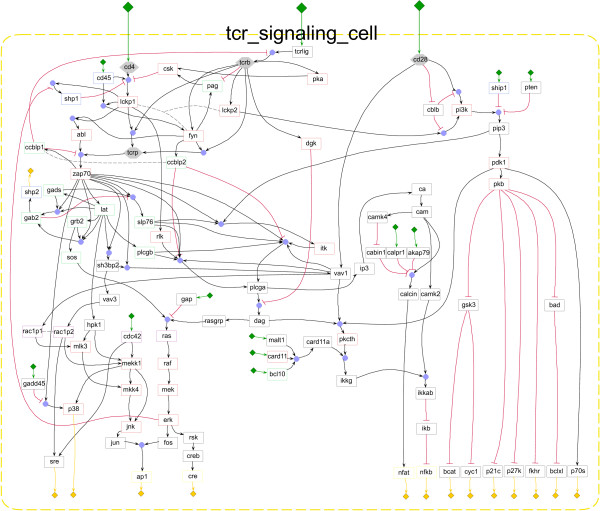
**Screenshot of a comprehensive logical model describing T-cell activation**. Screenshot of the logical model describing T-cell activation created in ProMoT. The model comprises 94 compound and 124 reactions.

In order to illustrate the applicability of our modeling environment to real, large networks, we have implemented, in ProMoT, a large model describing the signaling network governing T-cell activation (a previous version of the model described in [11]). The model comprises 94 species and 124 interactions (see Figure 6), and is, to the best of our knowledge, currently the largest logical model of a biological system. Note that we have used ProMoT's visualization properties described above, e.g. classifying the compounds to the different subclasses (kinase, adapter, etc.), which provides a map with this information visually coded (Figure 6).

## Discussion

In our group, there are currently several large models under development, including a model for T-cell signaling (see Figure 6), a model for the pro- and antiapoptotic signals controlling hepatocyte proliferation, and a detailed model of the cell cycle regulation in mammalian cells.

Thanks to ProMoT's modularity, we are developing parallely different submodels (e.g. EGF-dependent signaling, cell cycle regulation, and TNF-related signaling), which we will subsequently join into a larger model.

In order to represent the result of analyses of different tools directly in ProMoT's adaptive and interactive GUI, we plan to develop a common interface for data exchange.

Furthermore, we would like to develop, in agreement with the Systems Biology community and as part of the SBML initiative, a formalism for Boolean networks which facilitates the exchange and storage of models, as currently SBML allows for kinetics descriptions of biochemical networks.

## Conclusion

We have described a new framework to set up models of biochemical networks using a logical formalism. The flexibility of our approach, its consideration of the specific properties of signaling and regulatory processes and their requirements, makes it a useful tool for the Systems Biology community. Its applicability was demonstrated not only by a toy model, but also via the largest current logical model of a signaling network, comprising 94 compounds (see Figure 6).

## Availability and requirements

**Project name: **ProMoT

**Project home page: **

**Operating system(s): **The downloadable files run on Unix-derived operating systems (especially Linux). A Windows version is planned.

**Programming languages: **Java, Lisp

**Other requirements: **Java 1.4.2 or higher

**License: **GNU General Public Licence (GPL)

**Any restrictions to use by non-academics: **none

ProMoT is freely available at 

## Abbreviations

CNA = *CellNetAnalyzer*

CORBA = Common Object Request Broker Architecture

GinML = Gene Interaction Networks Markup Language

GUI = Graphical User Interface

ZUI = Zoomable User Interface

MDL = Model Definition Language

SBML = Systems Biology Markup Language

## Authors' contributions

JSR conceived the project, implemented the library with MG, tested it and set up the models. SM implemented the visualization methods. RH contributed to the setup of models (particularly the T-cell model) and the documentation. SK contributed to the export functions. EDG gave rise to fruitful discussions and coordinated the project. MG implemented the interpretation of the library in ProMoT and export of the models. All authors read and approved the final manuscript.

## Supplementary Material

Additional File 1 **ProMoT Tutorial**. A Tutorial explaining how to install ProMoT and how to set up and export logical models is attached. For updated versions check ProMoT's web page (see Availability and requirements section). Click here for file

Additional File 2 **ProMoT's source**. The source code of ProMoT is attached. ProMoT binaries, source, and ProMoT binaries plus all additional libraries (e.g. java) can be downloaded from ProMoT's web page (see Availability and requirements section). Click here for file
