# Supplementary material for: Visual setup of logical models of signaling and regulatory networks with ProMoT
Source: BMC Bioinformatics. 2006 Nov 17;7:506. doi: 10.1186/1471-2105-7-506 (PMC1665465; doi:10.1186/1471-2105-7-506)
Supplement: Additional File 1 — ProMoT Tutorial. A Tutorial explaining how to install ProMoT and how to set up and export logical models is attached. For updated versions check ProMoT's web page (see Availability and requirements section). [file 1471-2105-7-506-S1.pdf]

Tutorial for setting up logical models with ProMoT

Rebecca Hemenway, Julio Saez-Rodriguez,
Sebastian Mirschel, and Martin Ginkel

September 8, 2006

Max-Planck-Institute for Dynamics of Complex Technical Systems,
Sandtorstr. 1, 39106 Magdeburg, Germany.
mginkel@mpi-magdeburg.mpg.de

Contents

1 Introduction **3**

2 Preliminary: Getting ProMoT setup for the first use **3**
2.1 How do I install ProMoT? 3
2.2 How can I start ProMoT? 4
2.2.1 From the console 4
2.2.2 Using emacs 4

3 Getting Started: A look at the ProMoT GUI **4**

4 Building Your First Model: toymodel **6**
4.1 Creating Submodules 6
4.2 Saving Files 8
4.3 Loading ProMoT Library Elements 9
4.4 The Building Process 13

5 ProMoT Extras **18**
5.1 ProMoT Properties 18
5.2 Documentation in ProMoT 20

6 Visual Explorer **20**
6.1 Properties 21
6.2 Visual Scenarios 22

7 Exporting the Models **22**

8 Appendix **24**
8.1 Example Model 24
8.2 Visual Explorer Shortcuts and Modifiers 24

1 Introduction

The purpose of this tutorial is to introduce the ProMoT [1] interface to new users and to act as a guidance for becoming more familiar with the process of constructing logical models in ProMoT. ProMoT is also a tool used for constructing dynamic quantitative models and more information on this process can be found in a separate tutorial. This tutorial explains the basic components of ProMoT and uses the example of building a small simple modular model "toymodel" to enable one to become more comfortable with the process. For simplicities sake it does not dwell into the background architecture or design of the software, but is rather intended to be used as a jump start guide for the non-technically inclined.

More detailed documentation, particularly covering the application of *ProMoT* for dynamic models, can be found at:
http://www.mpi-magdeburg.mpg.de/projects/promot ProMoT manual, in the appendix "Getting Started".

2 Preliminary: Getting ProMoT setup for the first use

2.1 How do I install ProMoT?

Usually ProMoT is used from the editor Emacs. Therefore the line `(load "/usr/local/lisp/promot/src/emacs/promot")` must be inserted into your personal ~/.emacs[1].

1. Open a new terminal window like `kconsole` from the window manager. Usually there is a control panel in KDE and on this control panel you can find a screen-icon. Click on this icon **one** time, and you will get a terminal.

2. Type `emacs ~/.emacs`[1] and hit the return (enter) key. In all following commands we will assume implicitly that you hit return.

3. Move the cursor to the beginning of the file (it may be empty) and type: `(load "/usr/local/lisp/promot/src/emacs/promot")` . Save the file by the menu bar File > Save or by pressing the keys C-x C-s (this is the Emacs notation for Pressing Control and the "x" key at the same time followed by Control and the "s" key.) (Explanation: the `.emacs` is a startup-program of

[1] If the tilde '~' seems to be broken on a German (QWERTZU) Keyboard, it is possibly necessary to type the tilde key followed by an space ' '. Tilde is used in UNIX and Linux to refer to your personal home directory instead of typing the pathname all the time.

the text editor emacs. It is executed every time, emacs starts. By adding this line, some new entries are added to the menu of emacs to start ProMoT from there.)

4. Exit the Emacs by the menu bar File > Exit Emacs or by pressing C-x C-c.

At the next start of Emacs, ProMoT will be available.

2.2 How can I start ProMoT?

2.2.1 From the console

1. Use your Terminal window or start a new one as described in 2.1.
2. type "/usr/local/lisp/promot/scripts/promot" and ProMoT will get started.

2.2.2 Using emacs

1. Use your Terminal window or start a new one as described in 2.1.
2. Type `emacs` and emacs starts up.
3. Select in the menu-bar of Emacs: ProMoT>Start ProMoT.
4. The Browser window of ProMoT shows up.

Inside Emacs there are now two windows, showing useful information about the ProMoT system: `*promot-server*` and `*compilation*`. It is also possible to work interactively with the *promot-server*.

3 Getting Started: A look at the ProMoT GUI

Upon starting ProMoT the default (the bare necessities that compromise the program) ProMoT GUI (**g**raphical **u**ser **i**nterface: i.e. the windows, menus items, scroll bars, etc. that frames the main content that ProMoT presents as a working environment) loads the **browser**, which is the main window of ProMoT, see Figure 1. The browser contains four menus (**File**, **Class**, **Select**, and **Options**) with drop down selections allowing the user to find modeling entities, start graphical and textual editors as well as to load and save files.
There are two distinct areas of the ProMoT GUI, a **browser left panel** containing a **file tree** that shows the class hierarchy with all the modeling entities currently loaded and a **browser right panel** where, if desired, the currently selected classes .MDL source code can be previewed. (If you do not see the right panel we explain

how to view this in a bit). The file tree can be expanded and compacted by clicking the handle buttons to the left of each entity, where subclasses are shown in the sub-tree of the regarding super-classes. For example the default classes of ProMoT, as shown in Figure 1 are of the Modeling-entity and include:

1. behavioral-modeling-entity
   $>$ continuous-modeling-entity $>$ $>$ equation $>$ $>$ variable
   $>$ discrete-modeling-entity $>$ $>$ place $>$ $>$ transition

2. structural-modeling-entity
   $>$ module
   $>$ terminal

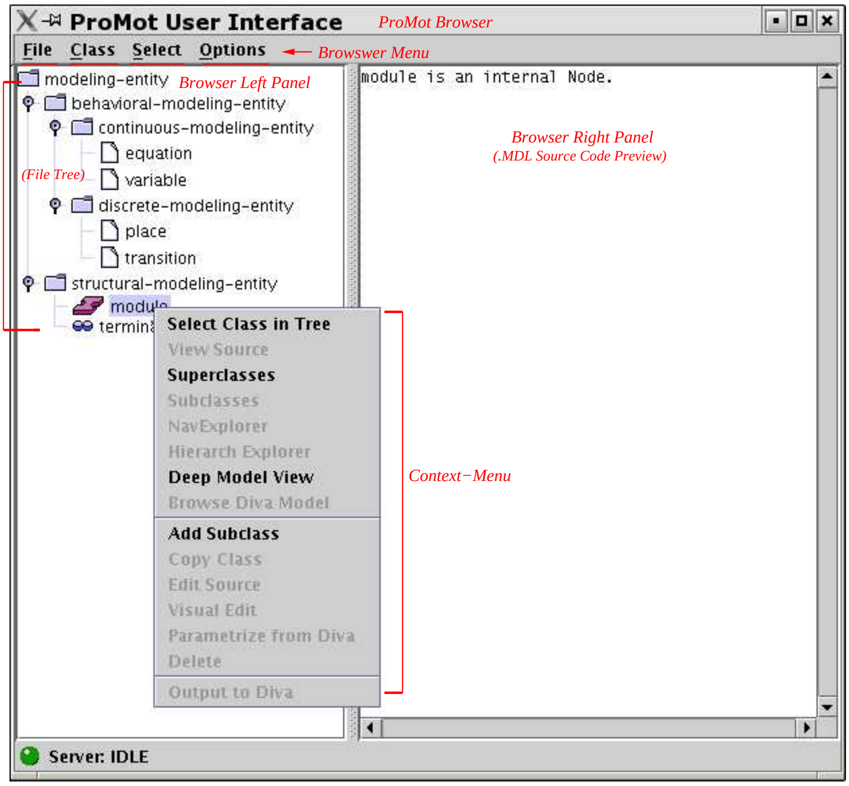

Figure 1: ProMoT Browser Window

For the first time user the most important modeling entities are located in the subtree of the structural-modeling-entity $>$ module. The file tree also has a **context-menu** (which can be viewed upon right-clicking) for each entity within it and is used for starting most editing actions. To go to the structural-modeling-entity $>$ module:

1. Click the handle button to the left of the folder icon of structural-modeling entity

2. Now there are two basic classes in view, **module** and **terminal** and no modeling-entities defined.

3. To view the context-menu right click on the modeling entity

4. The ProMoT browser enables the user to view either just the left file tree panel or both the left file tree panel an the right source code panel. To switch back and forth between modes click on the ProMoT browser menu > **Options** and select the check-box **Show Quickview**. With this option enabled it will preview the corresponding .MDL source code in the right panel of the ProMoT browser of the currently selected class. ProMoT models are written in .MDL, however this is all that is important for purposes here within.

4 Building Your First Model: toymodel

4.1 Creating Submodules

The following is a step-by-step tutorial to creating a simple hierarchical model using ProMoT. The name of the whole model will be **toymodel** and will contain the submodules (subclasses) **cell**, **whole_model**, and **nucleus**. which represent certain levels of a cell that are commonly examined when modeling a signal transduction pathway.

1. Start ProMoT and expand the **structural-modeling-entity** with the left handle button to view the **module** subclass.

2. Right click on **module** and click on **Add Subclass**, as seen here in Figure 2 . A window pop-ups asking to name the new subclass. Click within the text area of the pop-up window and type in the desired name for the model. (In our case type in: **toymodel**).

3. Click OK.

4. The naming pop-up window will disappear and a handle button will now appear next to the structural-modeling-entity > module in the ProMoT browser. Click on the handle button to expand the module tree. There you will see the subclass we just added as **"toymodel"**.

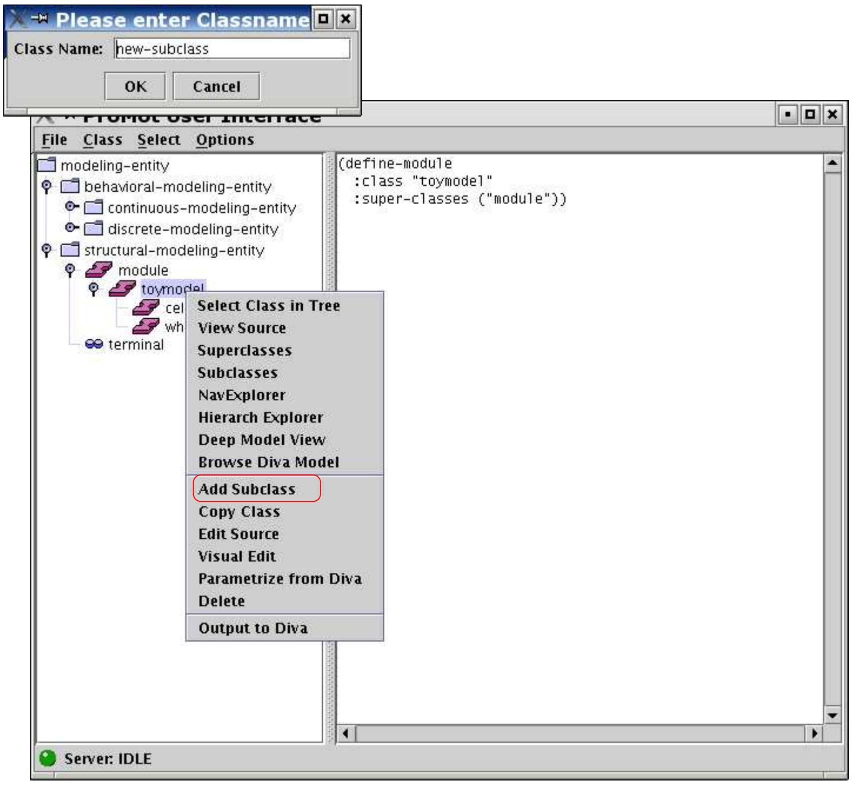

Figure 2: ProMoT Browser Window: Adding a Subclass

5. One of ProMoTs attributes is its ability to construct and handle models built in a modular style. Modules are attractive in modeling for purposes beyond the scope of this tutorial, but with this in mind let our first example include this approach and let us create the submodules as discussed above:

   (a) Right click on the **toymodel** and click on **Add Subclass** (as done in step 2 above, but under toymodel instead of module) but this time naming the subclass **cell**. Repeat this step to add the subclasses named **whole_model** and **nucleus** as well, being sure to right click and add the submodule under the **toymodel**.

4.2 Saving Files

Now let us save things before we get to the building part:

1. First it is recommended to make a folder where you will save all your files from ProMoT. In your home directory (or directory of your choice) make a folder called **promot**.

2. To make this directory:

   (a) Open a new shell terminal window

   (b) Make the directory by typing: **mkdir promot**

   (c) Change to that directory by typing: **cd promot**

3. Now that we have the main directory for all future model and simulation files let us create another directory for the toymodel example we are in the process of building. Using the same shell we just used to create the ProMoT directory, under the /promot]> directory make another directory called toymodel to save all the files associated with this example in:

   (a) in the /promot]> directory type: **mkdir toymodel**

   (b) change to that directory by typing': **cd toymodel**

4. Now that we have a directory for this project within our ProMoT folder now we are ready to save the files:

   (a) Click on the Superclass (i.e. **toymodel**) of all the submodules (i.e. **cell**, **nucleus**, **whole_model**) and go to the **Select** menu. From the Select menu chose: **Select** > **Select Subclasses**.

(b) Then go to: **File** > **Save Selected** and save the .mdl file in the toymodel directory just created named **toymodel.mdl**.

(c) Then select each subclass separately and save them each separately in the same directory. [2]

(d) One may also want to **delete** a class or **load** a different class into ProMoT. Since we have not started building a model yet, lets try this to become familiar with the process.

   i. To **delete** a model: (let us delete **cell** as an example) Click on the **cell** module in the **Browser left panel**, then right click on it and from the **Context-Menu** select **Delete**. The **cell** module will then dissappear from the ProMoT Browser but its corresponding .MDL source code will still be in the **toymodel** directory you saved it in previously. Now lets reload the file.

   ii. To **load** a model: From the ProMoT **Browser Menu** go to: File > Open and go to the directory toymodel directory and select the **cell** file and then click on **Open**. The cell module will then load back into the ProMoT user interface.

4.3 Loading ProMoT Library Elements

Before we begin the building process we need to load the building components of ProMoT, this is called the **ProMoT library:**
To do this you will need an initial library file from ProMoT [3] which includes logical components for building the models, such as the gates, compounds, and connecting elements.

1. Once you have this file load it into ProMoT by going to **File** > **Open** >> **struct-ana-library.mdl** (in our case this is the name of the library) and selecting the file and then clicking on **Open**.

2. Under the Browser left panel in the **module** the subclass **struct-library** will appear

3. Click on the handle button to the left of the **struct-library** to expand the library. There you will see subclasses of the library which are composed of the building blocks of ProMoT. (i.e. compound, gates, input, not, output, struct-adapter)

[2]Note: This step is not necessary but makes working with individual parts of the model easier until the whole thing is complete.

[3]typically at: /usr/local/lisp/promot/kb/SignalTransd/structure/

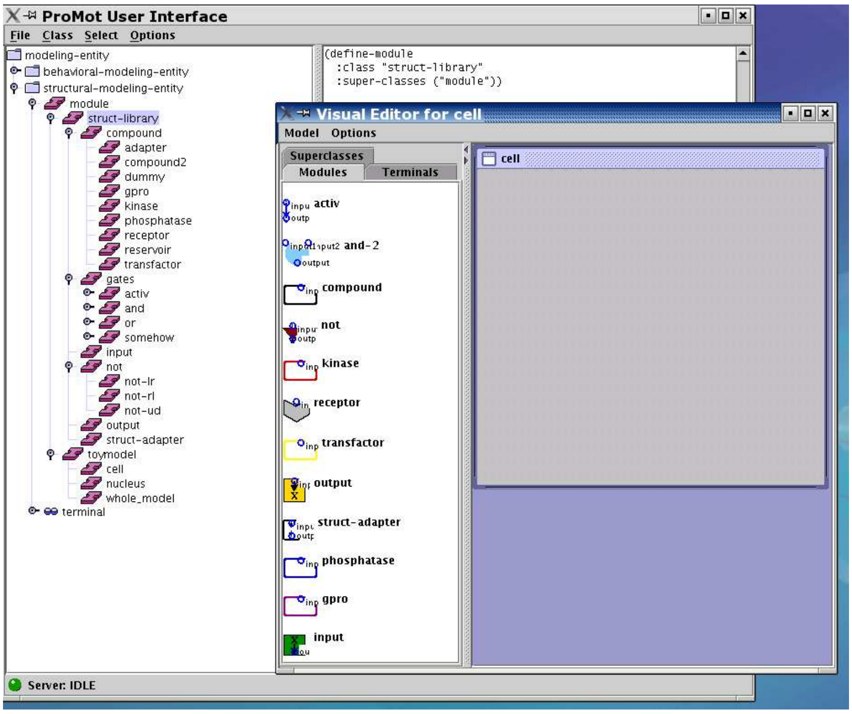

Figure 3: ProMoT Browser with Expanded Struct-Library and ProMoT Visual Editor for Cell Module

The main subclasses of the ProMoT library to build a logical model are as follows:

(a) **Compound:** To model logical states. Compound contains some common molecular classifications of certain species in a signaling network. These can be viewed by expanding the compound class by clicking on its corresponding handle button.(i.e. adapter (adapter protein), gpro (g-protein), kinase, phosphatase, receptor, transfactor (transcription factor) etc. Note that compounds can **not** be directly connected, and you need (at least) one element of the class gate between any 2 compounds. Examples will be discussed in section 4.4.

(b) **Gates:** Gates include the logical connecting components of the network for describing certain reactions and are based off of common boolean logic and operators.

   i. **Activ:** A single arc denoting the influence of one element on another is made via an **ACTIV** (activating gate); if the effect is negative, a **NOT** (see below and Fig.4(b)) should be additionally included.

   ii. **AND** Allow the connection of 2 or more compounds together and follow the "all or nothing" normal logic of an **AND** gate (see Fig.4(e)). We have built **AND** gates that allow up to 6 input species, however larger gates can also be built.

   iii. **OR:** A logic **OR** connection can easily be implemented using nodes (Fig.4(c); the inclusion of nodes is described in 4.4 and Fig. 6), alternatively an **OR** gate (Fig.4(c)) can be used to allow the connection of several species to one gate, if e.g. one wants to replace an AND gate by an OR gate.

   iv. **SOMEHOW** These gates act as a gate where the connection is not completely clear and allow to model incomplete truth tables. [2]

(c) **NOT** The **NOT** gate is used to represent an inhibitory effect on a compound. It must be used in place before any activating reaction gate (i.e. before an **Activ**, **AND**, **OR**, or **SOMEHOW** gate, see Figs.4(b),4(f)).

(d) **Input** Represents an input to a compound. All compounds that have no prior activating (or inhibiting) reaction to them must be connected to a input component (via an **Activ**).

(e) **Output** Represents the output of a compound

(f) **Struct Adapter** Allows to connect a node to a terminal

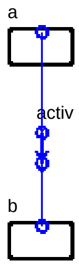

(a) ACTIV

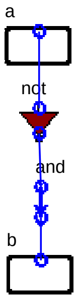

(b) NOT

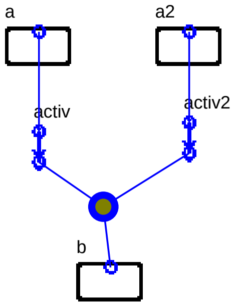

(c) OR (using a node)

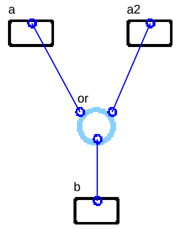

(d) OR (using a gate)

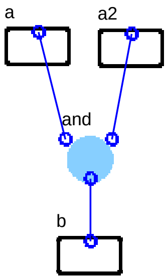

(e) AND

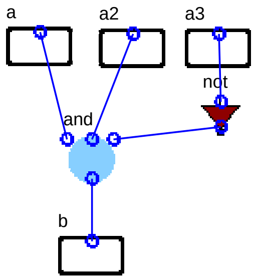

(f) Combination of activations and inhibitions in an AND gate

Figure 4: Implementation in *ProMoT* of basic logical connections.

4.4 The Building Process

We are going to be building the figure seen in Figure 5 which corresponds to “toymodel”. [3] This model represents a simple modular example of a cell signal transduction pathway. There are three main modular levels to toymodel:

(a) **whole_model** Represents the overlay of the whole cell and two extracellular inputs representing different possibilities of ligand binding to the cells receptors.

(b) **cell** Comprises the main inter-cellular signal transduction reactions that occur upon ligand binding to the cells receptor, down to (and including the nuclear phenomena)

(c) **nucleus** Represents the subsequent events that lead up to gene transcription, expression or proliferation

Let us begin the building process:

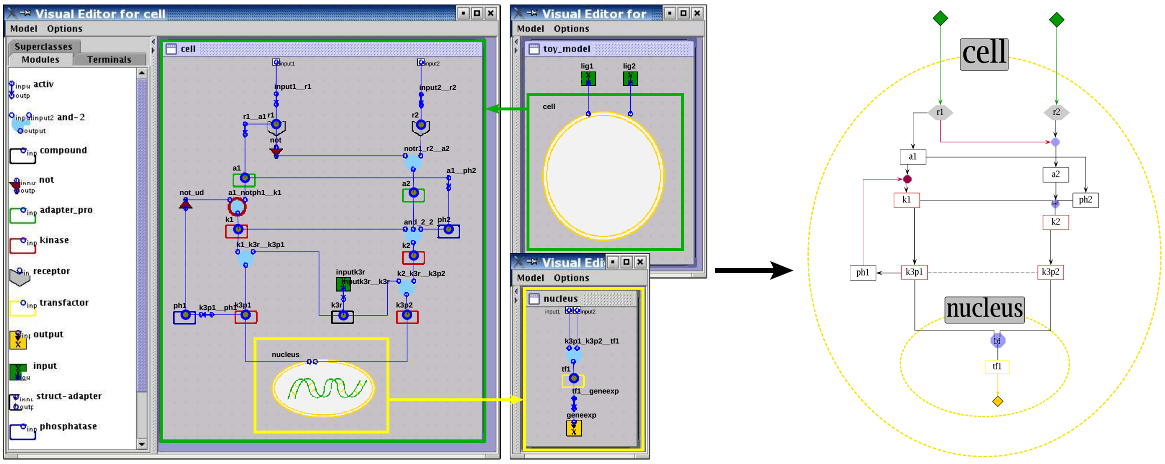

Figure 5: Toymodel described in [3]. See a larger version in the appendix 8

(a) Click on the **cell** module of the **toymodel** and once it is highlighted right-click on it to view the context-menu. Then select **Visual Edit**. This brings up a new window in ProMoT displaying the **ProMoT Visual Editor**, or the working panel of ProMoT.

(b) There are two main areas to the **ProMoT Visual Editor**

   i. The left area where there are tabs for: Modules, Terminals and Superclasses.
   ii. The canvas or template area on the right where the model is built.

(c) Click on the Modules tab. There you should see some of the building components from the struct-library file. But one may want to add more elements here for easy use. To do that:

   i. From the ProMoT Browser Left Panel simply drag and drop the desired component into the Modular tab area of the Visual Editor.
   ii. If you would like that component to always load upon opening of the Visual Editor by default, click on that component within the Visual Editor Module, then right-click and select **Default Class**.
   iii. If you want to delete a certain component from loading by default into the Visual Editor select the component, right-click and select **Remove Default**
   iv. Finally, if you want to delete the component from the Modular tab area altogether, select the component, right click and select **Delete**

(d) Now, to build the model you just drag and drop components from the module tab area into the canvas part of the ProMoT Visual Editor. For instance, in our example of the cell module we have a receptor named **r1**. To add this receptor:

   i. Click on the receptor compound in the module tab and drag it onto the cell canvas.
   ii. To rename the component, within the model right click on the component and select **properties**. Under **Slot name** type in the desired name (in our case **r1** and click on **Set** then **Close**.

(e) Next connect compounds together in the network by different gates. Let us for example make the connection from **r1** $\rightarrow$ **a1**:

   i. Drag a **adapter** (protein) onto the canvas and rename it to **a1**
   ii. Drag a **activ** onto the canvas and rename it **r1__a1**[4]
   iii. Now connect the input terminal of the activ gate to the terminal connection (the little blue circle) in r1 and then connect the output terminal of the activ gate to the terminal connection (little blue circle) of a1.
   iv. Our first connection has been made. You should have something similar to 6(a).

[4] the names given to the gates can be chosen freely and have no importance

(f) Now as one can see in Figure 5 more than one compound is attached to r1. Therefore we need to make it possible to add more than one connection to its terminal. To do this:

   i. Click on the terminal connection (the blue circle) of the compound (here r1) or on the link (blue line) connecting it to the active.

   ii. Then right click and select **Insert Node**. You should have something similar to 6(b).

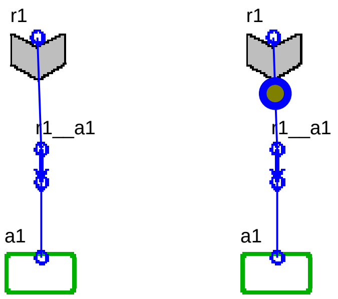

(a) r1 activates a1

(b) addition of a node to add further connections

Figure 6: First steps in setting up the toy model. The final model is displayed in 7.

(g) You can move any of the elements around on the canvas, for visually more appealing positions, by selecting it and moving it with either your mouse, or with the arrow keys from your keyboard.

(h) Now let us add the second receptor, **r2**, **a2**, and the **notr1_r2__a2** AND gate:

   i. Drag a **not** gate onto the canvas. [5]

   ii. Drag a **and-2** gate onto the canvas and name it **notr1_r2__a2**

   iii. Drag another **adapter** onto the canvas and name it **a2**

   iv. Drag another **receptor** onto the canvas and name it **r2**

(i) Connect them together by clicking **r2** and then click and drag your mouse from the **r2** terminal connection to one of the two inputs of the **and-2** gate.

[5] as for the gates, the name of the not elements have no relevance

(j) As mentioned before the **not** gate is used to represent an inhibitory effect. Click on the input of the **not** and then click on its terminal connector and click and drag to connect it to the node of **r1**. Then click on the **not** again and click on the output terminal and click and drag it to connect to the other input of the **and-2** gate.

(k) Now click on the output terminal of the **and-2** gate and then click and drag this to the **a2** terminal connector.

(l) Continue building the cell model in this fashion until it looks like Figure 7 below, remembering to name the elements before connecting them and also adding nodes to compounds that you would like to add more than one connection to. Also, be sure to **save** the model through the Visual Editor periodically by selecting **Model** > **Save**.

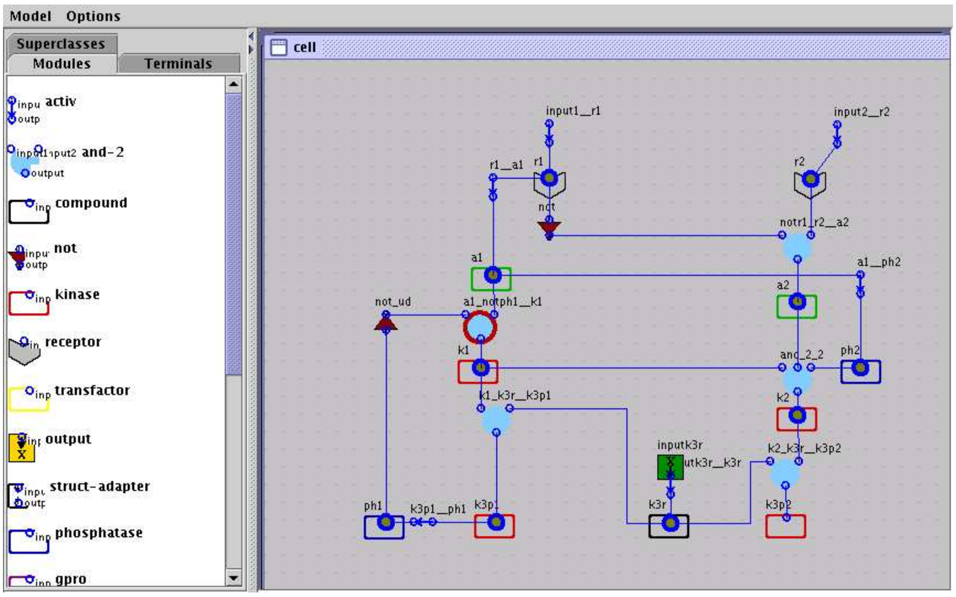

Figure 7: Building a Model: ProMoT Visual Editor: Cell Module

(m) Now you need to add the input and output components to the model. If this were a single (non-modular) model one could use just the **input** and **output** library components, however because we want our input components to come from another module we need to tell ProMoT this and add a terminal structure input. To do this:

   i. Click on the input1_r1 **activ** gate and then right click and select **Terminal Connections** > **Propagate input**. Rename the terminal connection to **input1**.

ii. When you have added both inputs to the model save the model through the Visual Editor as explained earlier and then close it by clicking on the **X** on the upper-right hand corner. Do not worry about the connection to the **Nucleus** Module at this point (we will get back to that).

(n) Now back in the **ProMoT Browser** select the **cell** module and **save** it to its own file by selecting **File** > **Save Selected** and overwriting the cell.mdl file you first created. This makes it so, later in time, you can load the file independently if so desired.

(o) Next, select the **nucleus** module and open it in the **Visual Editor** as you had previously done with the **cell** module.

(p) Build the model as done in previous steps according to the **nucleus** module seen in Figure 5 being sure to save the file in both the Visual Editor and the ProMoT Browser.

(q) Next, click on and open **whole_model** in the **Visual Editor** and add the two input elements **lig1** and **lig2**.

(r) Now we need to connect the **cell** module to this **whole_model** module. To do this:

i. Within the **ProMoT Browser** click on the **cell** module and drag and drop it into the Modules tab area of the **Visual Editor** (as done previously to add library components to the Visual Editor).

ii. Then just as you build the model, drag the cell onto the canvas and connect the input terminal connection from **lig1** to the first input of the **cell** and the input terminal connection from **lig2** to the second input of the **cell**.

iii. **Save** the file in the **Visual Editor** as well as in the **ProMoT Browser**. [6] You will not see the circular icon of the cell that is in Figure 5, but rather just a white box with the modular name **cell** typed in blue font on its inside. Until you design your own icon to implement into the code this is what it will look like, and this topic is beyond the scope of this tutorial.

(s) Next we need to add the **nucleus** module to the **cell** module.

(t) As done before, open the **cell** module to the **Visual Editor** from the **ProMoT Browser**.

[6] Note:

(u) Then drag the **nucleus** module from the **ProMoT Browser** into the Modules tab area of the **Visual Editor**.

(v) Then drag the **nucleus** module into the **cell** canvas and connect the corresponding connections to its input terminal connections. (In our case: **k3p2** to **nucleus** input2 and **k3p1** to **nucleus** input1).

(w) **Save** the file in both the **Visual Editor** and **ProMoT Browser**.

(x) Finally, save the whole superclass **toy_model** again with all subclasses selected. (Click on the **toy_model**, click on **Select** > **Select Subclasses**, and then click on **File** > **Save Selected** and overwrite the toy_model.mdl file saved previously.

5 ProMoT Extras

5.1 ProMoT Properties

While building logical models there are different properties that are often useful for describing the characteristics of the model. ProMoT has implemented some of these concepts into properties of the model, these properties are considered "variables." These properties include the following:

1. **time:**This variable is the parameter determining the time-scale for the reactions. These can be labels either 1,2, or 3; 1 being the default value and earliest (or fastest) time event and 3 being the slowest event.

2. **defval:** This is to set the logical default value of a compound, and in this case since we are using logical models they can be set as either 1 or 0. By default ProMoT sets the default value to nothing.

3. **conf:** Degree of Confidence. This variable is used to set a level of how confident or well supported a certain reaction is. One being the most established.

4. **a:** Level of activation. This parameter is default 1, and can be modified to encode multilevel logical operations. For example, in the toymodel (see Fig. 5), tf1 reaches a level 2 if both k3p1 and k3p2 are active. This is set up in *ProMoT* via a gate of the class AND where the output has **a**= 2 (see Fig. 8).

These properties can be changed in the Visual Editor of ProMoT by selecting the specific reaction gate, then right-clicking on it and selecting "Edit Variables"

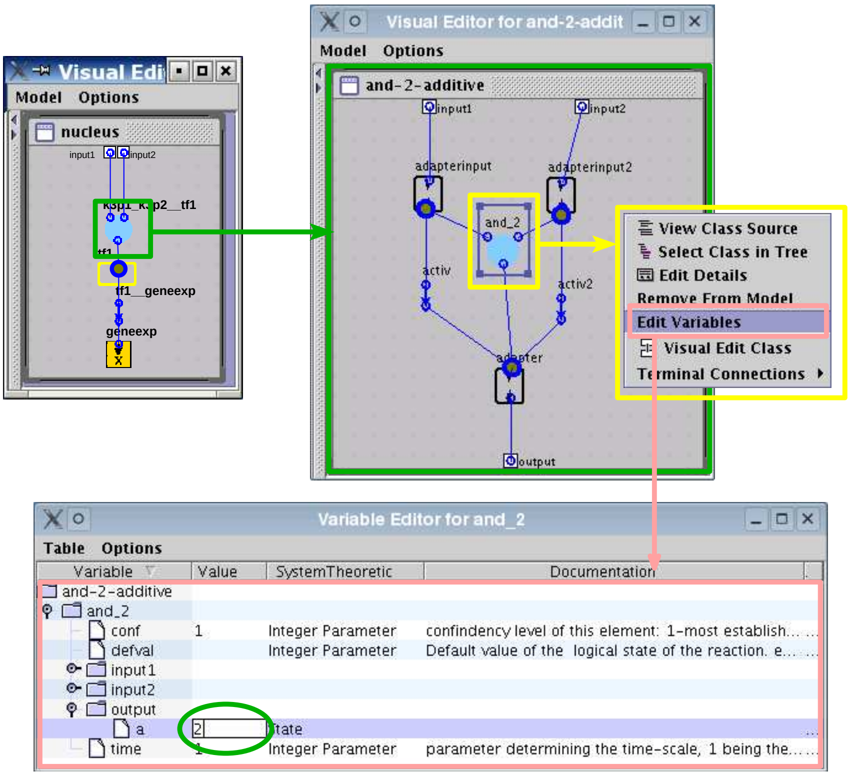

Figure 8: Properties like the *multilevel* variable a can be edited in the variable editor.

5.2 Documentation in ProMoT

The construction of signaling networks often leads to large complex models. One of the most important things to do while building these models is to keep track of the references of where the data supporting the architecture of these models is coming from. ProMoT allows this to be implemented while building the model in the Visual Editor.

1. When in the Visual Editor select the reaction of your choice
2. Right-click on the reaction and choose **Properties**
3. Under the Slot class selection area there is an area labeled **Documentation**, click so the cursor becomes active there and type the desired documentation
4. Then click on **Set**
5. Then click on **Close**

6 Visual Explorer

The *Visual Explorer* is an additional entity of *ProMoT* . To get to the *Visual Explorer*:

- Within the *ProMoT* **Browser Left Panel** select the model of your choice.
- Right-click on the model and from the **Context-Menu** select **Visual Explorer**.
- While loading an information window appears.
- After a certain time the *Visual Explorer* will pop-up (depends on the size of the network).

The Visual Explorer is used for creating and manipulating different graphical representations of a network. For this purpose, the concept of *Visual Scenarios* are introduced. A visual scenario is a set of visual mapping functions for changing visual properties of the network (layout, display) and defines adequate user interactions. Therewith, convenient and fast switching between different visual settings is feasible. Almost all visual properties of the network and its parts can be altered, e.g. color, size, line type.
For setup a visual scenario:

- Middle-click on the model within the Visual Explorer and from the **Context-Menu** select **Edit Scenario. . .**
- Select a new scenario in the **Popup-Menu**.
- Or alter the current scenario by clicking on **Define**.
- There is also a **Popup-Menu** at the bottom line of the Visual Explorer window where a new scenario can be choosen.

6.1 Properties

Properties like the *multilevel* variable or the *visibility* variable can be edited in the variable editor (how to use the variable editor see . . .). The parameter `a` (level of activation), is default 1 (level 1), and can be modified to encode multilevel logical operations. The parameter `ContentVisible` (visibility of the content), is default 1 (visible), and can be used . . . .

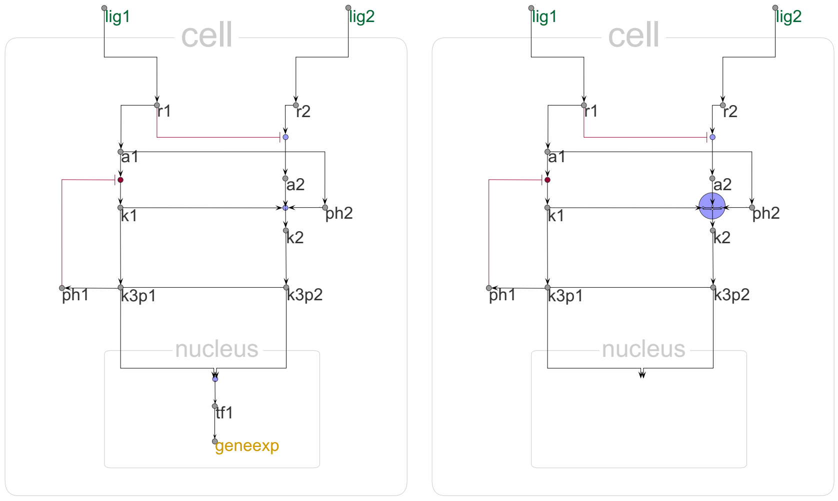

Figure 9: Alternative visual scenario towards a more abstract representation. Proteins have a uniform color, connection lines are orthogonalized, and the border of `cell` and `nucleus` are de-emphazised to draw the attention to both logical operations and connections. Additionally, to illustrate the `visibility` property, in the right figure the visibility of the module `nucleus` has been set to 0 and that of the logical gate `and_2_2` to 1 (for illustration purposes the gate `and_2_2` is displayed enlarged).

6.2 Visual Scenarios

In the visual explorer different scenarios are predefined, e.g. “Logical Network”, “Connectivity” or “Semantic Distance”. These are common scenarios that have the following functionalities

- **Logical Network**
  . . . .
- **Connectivity**
  Each entity is ranked by a connectivity value based on the link structure (incoming and outgoing links). Hence, connectivity is a property of graphs and is used for analysis in graph theory. Here, the connectivity value is encoded by a color gradient.
- **Semantic Distance**
  Semantic distance means the distance from a focused entity to another entity in the network structure. Semantic distance is encoded by color. This scenario is interactive (user has to set the focus entity and eventually a second focus entity).

Furthermore, the *Visual Explorer* is more appealing for publication, exportation, and printing. For an overview of interaction pattern, shortcuts and modifiers, please refer to Table 1 in the appendix.

7 Exporting the Models

There is currently no standard for logical networks (note that SBML only supports kinetic models). There has been recently an initiative to define a standard for boolean networks (GinML, see [4]) which is, however, still not established. Therefore, we have so far only implemented two formats: one for *CellNetAnalyzer* (CNA) [2] and one in an alternative matlab formalism. New formats, however, can be easily implemented.

To export the logical ProMoT model with its corresponding graphic file and mathematical components,open the model of your choice in the Visual Explorer as discussed previously. Then, right click on the model and select “Output Logical Model” from the menu, choose the format, and save it to the directory of your choice. To get *CellNetAnalyzer*, go to
http://www.mpi-magdeburg.mpg.de/projects/cna/cna.html.
In order to perform analyses as those from[2], do **not** to check the “Export Boundary Compounds”. Besides, the “Simples Names” option will export quite compact names.

References

[1] M. Ginkel, A. Kremling, T. Nutsch, R. Rehner, and E. D. Gilles. Modular modeling of cellular systems with promot/diva. *Bioinformatics*, 19(9):1169–1176, June 2003. http://bioinformatics.oxfordjournals.org/cgi/content/abstract/19/9/1169.

[2] S. Klamt, J. Saez-Rodriguez, J. Lindquist, L. Simeoni, and E. D. Gilles. A methodology for the structural and functional analysis of signaling and regulatory networks. *BMC Bioinformatics*, 7(56), 2006. http://www.biomedcentral.com/1471-2105/7/56,

[3] J. Saez-Rodriguez, S. Mirschel, R. Hemenway, S. Klamt, E. D. Gilles, and M. Ginkel. Visual set-up of logical models of signaling and regulatory networks with promot. submitted.

[4] A. Gonzalez and A. Naldi and L. Sanchez and D. Thieffry and C. Chaouiya GINsim: A software suite for the qualitative modelling, simulation and analysis of regulatory networks *Biosystems* 2,91-,100,2006.

8 Appendix

8.1 Example Model

8.2 Visual Explorer Shortcuts and Modifiers

Operation	*Shortcut*	*Description*
**Menus**		
Open global menu	Click middle button	Opens the global context menu
Open node menu	Click right button on entity	Opens the node context menu
Open edge menu	Click right button on entity	Opens the edge context menu
**Navigation Mode: Pan+Zoom**		
Zoom in	Hold down left button + Drag right	Zoom in the network
Zoom out	Hold down left button + Drag left	Zoom out the network
Pan	Hold down left button + Shift + Drag	Move the network
**Navigation Mode: Hierarchy Zoom**		
Zoom in	Click left button on entity	Zoom to focused entity in hierarchical steps
Zoom out	Click left button on entity	Zoom to focused entity in hierarchical steps
Fit network on screen	Click left button + Shift	Zoom out the whole network to fit on screen
**Navigation Mode: FishEye Zoom**		
Zoom in	Click left button on entity	
Zoom out	Click left button on entity + Shift	
**Navigation Mode: Focus Zoom**		
Zoom to	Click left button on entity	Minimal zoom to focused entity
**Navigation Mode: Detail+Overview**		
Pan	Click left button on gray box + Drag	Move the network
Zoom	Click left button on handle of gray box + Drag	Zoom in or out the network
**Editor Mode: Node**		
Move	Click left button on entity + Drag	Move node inside bounds of parent node
Resize (all)	Click left button on handle + Drag	
Resize (border)	Click left button on handle + Shift + Drag	
**Editor Mode: Edge**		
Move handle	Click left button on handle + Drag	Move edge handle
**Scenario Mode: Semantic Distance**		
Show Sem Dist	Click left button on entity	Show Semantic Distance for focused entity
Shortest Path (two)	Click left button on two entities + Shift	
Shortest Path (all)	Click left button on two entities + Ctrl	

Table 1: Shortcuts and modifiers used in the *Visual Explorer*.

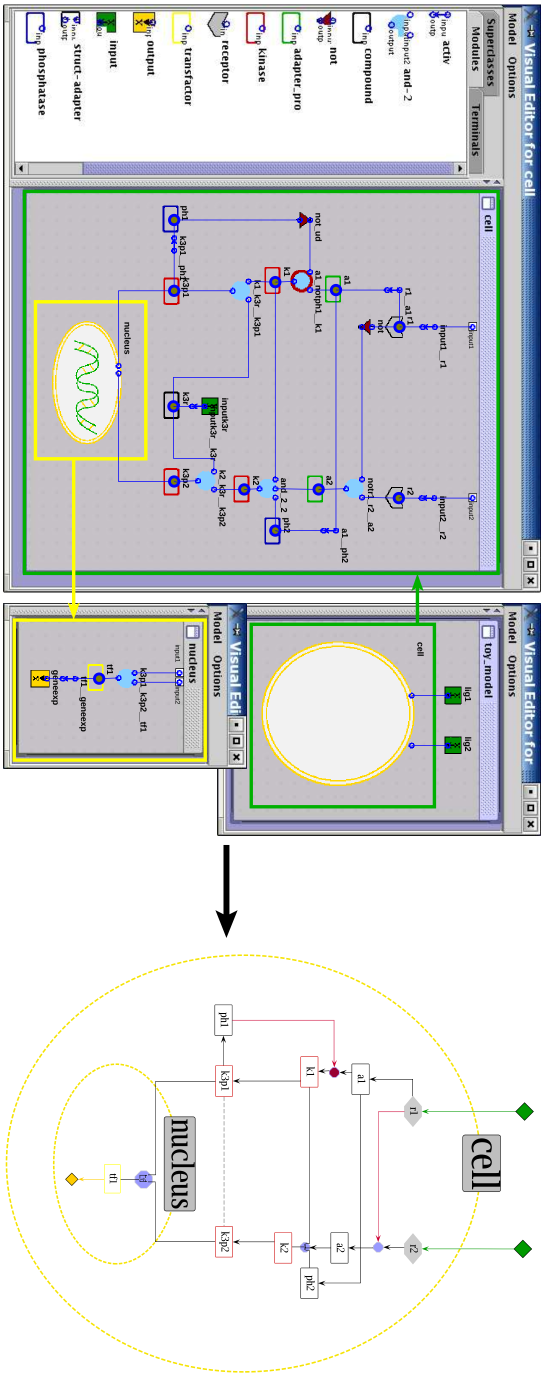

Figure 10: Toymodel described in [3].
