# Supplementary material for: Visual setup of logical models of signaling and regulatory networks with ProMoT
Source: BMC Bioinformatics. 2006 Nov 17;7:506. doi: 10.1186/1471-2105-7-506 (PMC1665465; doi:10.1186/1471-2105-7-506)
Supplement: Additional File 2 — ProMoT's source. The source code of ProMoT is attached. ProMoT binaries, source, and ProMoT binaries plus all additional libraries (e.g. java) can be downloaded from ProMoT's web page (see Availability and requirements section). [file 1471-2105-7-506-S2.bz2 › Promot/xml/code/atn-parser/clifs/README.htm]

untitled


### CLIFS

this defines only the housekeeping clases and functions from the CLIFS system
for use with the ATN-BNF parser.

---

#### loading

the files integrated in the parser defsystem.

---

#### usage

the inferencsystem an units classes serve as abstract classes for atn compiler
classes.

---

#### credits

the CLIFS library is the work of benno biewer.

---

©setf.de 2001
