# Supplementary material for: Visual setup of logical models of signaling and regulatory networks with ProMoT
Source: BMC Bioinformatics. 2006 Nov 17;7:506. doi: 10.1186/1471-2105-7-506 (PMC1665465; doi:10.1186/1471-2105-7-506)
Supplement: Additional File 2 — ProMoT's source. The source code of ProMoT is attached. ProMoT binaries, source, and ProMoT binaries plus all additional libraries (e.g. java) can be downloaded from ProMoT's web page (see Availability and requirements section). [file 1471-2105-7-506-S2.bz2 › Promot/xml/code/atn-parser/README.htm]

untitled


### ATN - BNF compiler

the BNFP module implements an ATN-based parser generator. the generator accepts
EBNF syntax specifications and generates either lisp or java source for the specified
language.

---

#### loading

The file **`defSystem.lisp`** loads the module.

The java generator is included, and will be loaded only if the feature **`BNFP-JAVA`**
is defined.

---

#### usage

The module is defined in the package "BNF-PARSER" ("BNFP")".
all interface functions are defined in and exported from this package. it has two
mode of usage: **generation** and **production**. in either mode, a grammar
source file serves as the basis for processing. it comprises the BNF text description
and effects the translation.

generation
:   In order to develop a parser for an application, the complete BNFP package must
    be loaded so as to include the atn->lisp translator. the function **`COMPILE-ATN-SYSTEM`**
    translates a BNF description into an ATN instance and translates that ATN into a
    definition for lisp function. if the atn compiler is invoked with the setting **`:EXECUTE
    t`** this function definition is evaluated in the active runtime environment.
    if the atn compiler is invoked with the setting **`:COMPILE t`**`,`
    then the definition is stored in an external file and compiled from there. this file
    is specified with the **`:PATHNAME`** argument to the compiler. by
    default, a file named after the grammar is created in the relative directory **`ATN-LIB`**.  
    the first time a grammar is compiled, the several additional files are copied into
    the destination directory and compiled. the generated parser source includes steps
    to load these into the eventual runtime environment.

    production
    :   In order to use a parser within an application, load the file emitted during
        the generation step and the runtime support file.

An application could be coded as follows. this assumes a logical host definition
(*`host`***:**) which includes a designation for the location
of the atn parser (*`host`***:***`ATN`***;**)

> ```
> (defparameter *phrase-bnf*
>
>   "phrase ::= (term | (term and phrase) | (term or phrase) | ' ')*
>
>    term ::= text+
>
>    comma ::= ','
>
>    or ::= '|' | '+'
>
>    and ::= '&' | '*'
>
>    text ::= [#x30-#x39] | [#x61-#x7a] | [#x41-#x5a]"
>
>   "the bnf for subject text")
> ```
>
> ```
> ;; this convenience function is used to transform string input to a vector
>
> (defun phrase-tokenizer (s) (concatenate 'vector s))
>
>
> #-ATN-COMPILER
>
> (eval-when (:compile-toplevel :execute)
>
>   ;; make sure the grammar compiler is present.
>
>   (load "host:ATN;defSystem.lisp")
>
>   (use-package "BNFP"))
> ```
>
> ```
> (eval-when (:compile-toplevel :execute)
>
>   (compile-atn-system *phrase-bnf* :compile t
>
>                      :pathname "host:ATN-LIB;phrase-grammar.lisp"))
>
>
> (eval-when (:load-toplevel)
>
>   (load "host:ATN-LIB;phrase-grammar"))
>
>
> (eval-when (:execute)
>
>  (compile-atn-system *phrase-bnf* :execute t))
> ```

once loaded or executed the parser would be used as follows

> `(phrase-parser "s&a" :atn-trace t)`

---

#### credits

The parser derives from work with benno biewer together at mecom gmbh on an ATN-based
parser for java.

#### ---

#### license

This library is free software; you can redistribute it and/or modify it under
the terms of the GNU Lesser General Public License as published by the Free Software
Foundation; either version 2.1 of the License, or (at your option) any later version.

This library is distributed in the hope that it will be useful, but WITHOUT ANY
WARRANTY; without even the implied warranty of MERCHANTABILITY or FITNESS FOR A PARTICULAR
PURPOSE. See the GNU Lesser General Public License for more details.  
  
You should have received a copy of the GNU Lesser General
Public License along with this library; if not, write to the Free
Software Foundation, Inc., 59 Temple Place, Suite 330, Boston, MA 02111-1307
USA  


---

©setf.de 2001
