# Supplementary material for: Visual setup of logical models of signaling and regulatory networks with ProMoT
Source: BMC Bioinformatics. 2006 Nov 17;7:506. doi: 10.1186/1471-2105-7-506 (PMC1665465; doi:10.1186/1471-2105-7-506)
Supplement: Additional File 2 — ProMoT's source. The source code of ProMoT is attached. ProMoT binaries, source, and ProMoT binaries plus all additional libraries (e.g. java) can be downloaded from ProMoT's web page (see Availability and requirements section). [file 1471-2105-7-506-S2.bz2 › Promot/xml/demos/saxandsoap/TODO.htm]

19-sep-01
Stanley Knutson (knewt@alum.mit.edu)
Various items remaining here:
- the sax-basics file still has some unused stuff
- the CDATA handling in the sax api is not tested
[Also, the underlying CL-XML does not pass data in reasonable chunks
yet]. Not an issue for SOAP.
- There is no SOAP 'writer' yet.
- I've not yet verified this with many soap files.
The true test will be interoperation with Apache soap
and that requires an HTTP 1.1 server
- Numerous bits of SOAP are not implemented:
TYPES:
arrayCoordinate
arrays with offset
decimal type
times [there are several kinds: time, date etc, duration, instances]
uri reference
binary
long
short
byte
unsigned-x types
PROTOCOL:
ID, IDREF, Entity, Notation,
The envelope and body should have distinct classes
so the headers can be properly handled [or generated]
There needs to be a 'fault' class
Various bits in the Apache Java API's would make sense to be copied
in some fashion
There is no support for UTF-8, just ISO-8859-1 right now
[this may be fixable in the lower level xml parser]
