# Supplementary material for: Visual setup of logical models of signaling and regulatory networks with ProMoT
Source: BMC Bioinformatics. 2006 Nov 17;7:506. doi: 10.1186/1471-2105-7-506 (PMC1665465; doi:10.1186/1471-2105-7-506)
Supplement: Additional File 2 — ProMoT's source. The source code of ProMoT is attached. ProMoT binaries, source, and ProMoT binaries plus all additional libraries (e.g. java) can be downloaded from ProMoT's web page (see Availability and requirements section). [file 1471-2105-7-506-S2.bz2 › Promot/xml/demos/saxandsoap/README.htm]

## SAX api

This directory has the SAX api for lisp parsing of XML files.

The majority of the code here written by Stanley Knutson
(knewt@alum.mit.edu)

Some of code in sax-basics came from Wilbur-RDF project
However, it has been modified significantly to use the underlying
CL-XML parser from cl-xml.org

This is a "work in progress" since there is not yet any tools for
writing SOAP.

The top-level function is sax:soap-xml-parse.
