# Supplementary material for: Visual setup of logical models of signaling and regulatory networks with ProMoT
Source: BMC Bioinformatics. 2006 Nov 17;7:506. doi: 10.1186/1471-2105-7-506 (PMC1665465; doi:10.1186/1471-2105-7-506)
Supplement: Additional File 2 — ProMoT's source. The source code of ProMoT is attached. ProMoT binaries, source, and ProMoT binaries plus all additional libraries (e.g. java) can be downloaded from ProMoT's web page (see Availability and requirements section). [file 1471-2105-7-506-S2.bz2 › Promot/xml/W3C-copyrightsoft-19980720.htm]

W3C IPR SOFTWARE NOTICE


# W3C^®^ SOFTWARE NOTICE AND LICENSE

### Copyright © 1994-2001 World Wide Web Consortium, (Massachusetts Institute of Technology, Institut National de Recherche en Informatique et en Automatique, Keio University). All Rights Reserved. http://www.w3.org/Consortium/Legal/

This W3C work (including software, documents, or other related
items) is being provided by the copyright holders under the
following license. By obtaining, using and/or copying this work,
you (the licensee) agree that you have read, understood, and will
comply with the following terms and conditions:

Permission to use, copy, modify, and distribute this software
and its documentation, with or without modification,  for any
purpose and without fee or royalty is hereby granted, provided that
you include the following on ALL copies of the software and
documentation or portions thereof, including modifications, that
you make:

1. The full text of this NOTICE in a location viewable to users of
   the redistributed or derivative work.
2. Any pre-existing intellectual property disclaimers, notices, or
   terms and conditions. If none exist, a short notice of the
   following form (hypertext is preferred, text is permitted) should
   be used within the body of any redistributed or derivative code:
   "Copyright © [$date-of-software] 
   World Wide Web Consortium, (Massachusetts Institute of
   Technology, Institut National de
   Recherche en Informatique et en Automatique, Keio University). All Rights Reserved.
   http://www.w3.org/Consortium/Legal/"
3. Notice of any changes or modifications to the W3C files,
   including the date changes were made. (We recommend you provide
   URIs to the location from which the code is derived.)

THIS SOFTWARE AND DOCUMENTATION IS PROVIDED "AS IS," AND
COPYRIGHT HOLDERS MAKE NO REPRESENTATIONS OR WARRANTIES, EXPRESS OR
IMPLIED, INCLUDING BUT NOT LIMITED TO, WARRANTIES OF
MERCHANTABILITY OR FITNESS FOR ANY PARTICULAR PURPOSE OR THAT THE
USE OF THE SOFTWARE OR DOCUMENTATION WILL NOT INFRINGE ANY THIRD
PARTY PATENTS, COPYRIGHTS, TRADEMARKS OR OTHER RIGHTS.

COPYRIGHT HOLDERS WILL NOT BE LIABLE FOR ANY DIRECT, INDIRECT,
SPECIAL OR CONSEQUENTIAL DAMAGES ARISING OUT OF ANY USE OF THE
SOFTWARE OR DOCUMENTATION.

The name and trademarks of copyright holders may NOT be used in
advertising or publicity pertaining to the software without
specific, written prior permission. Title to copyright in this
software and any associated documentation will at all times remain
with copyright holders.

\_\_\_\_\_\_\_\_\_\_\_\_\_\_\_\_\_\_\_\_\_\_\_\_\_\_\_\_\_\_\_\_\_\_\_\_

This formulation of W3C's notice and license became active on
August 14 1998 so as to improve compatibility with GPL. This
version ensures that W3C software licensing terms are no more
restrictive than GPL and consequently W3C software may be
distributed in GPL packages. See the older formulation for the
policy prior to this date. Please see our 
Copyright FAQ for common questions about using materials from
our site, including specific terms and conditions for packages like
libwww, Amaya, and Jigsaw. Other questions about this notice can be
directed to 
site-policy@w3.org.

webmaster  
(last updated $Date: 2001/06/01 21:40:06 $)
