# Supplementary material for: Visual setup of logical models of signaling and regulatory networks with ProMoT
Source: BMC Bioinformatics. 2006 Nov 17;7:506. doi: 10.1186/1471-2105-7-506 (PMC1665465; doi:10.1186/1471-2105-7-506)
Supplement: Additional File 2 — ProMoT's source. The source code of ProMoT is attached. ProMoT binaries, source, and ProMoT binaries plus all additional libraries (e.g. java) can be downloaded from ProMoT's web page (see Availability and requirements section). [file 1471-2105-7-506-S2.bz2 › Promot/promot/src/gui/org/apache/log4j/xml/examples/package.html]

 


Example usage of log4j with XML (including source code).

This package's shows how log4j can be used with an XML
configuration file.

See source code of XMLSample.java showing how to
configure with an XML file. Sample XML files
sample1.xml,
sample2.xml,
sample3.xml,
sample4.xml,
sample5.xml are provided.

---


Last modified: Wed Apr 12 22:27:25 MDT 2000
