# Supplementary material for: Visual setup of logical models of signaling and regulatory networks with ProMoT
Source: BMC Bioinformatics. 2006 Nov 17;7:506. doi: 10.1186/1471-2105-7-506 (PMC1665465; doi:10.1186/1471-2105-7-506)
Supplement: Additional File 2 — ProMoT's source. The source code of ProMoT is attached. ProMoT binaries, source, and ProMoT binaries plus all additional libraries (e.g. java) can be downloaded from ProMoT's web page (see Availability and requirements section). [file 1471-2105-7-506-S2.bz2 › Promot/promot/src/gui/org/apache/log4j/chainsaw/package.html]

Chainsaw Tool


Chainsaw is a GUI log viewer and filter for the log4j
package. By default it listens for LoggingEvent objects sent using
the SocketAppender and
displays them in a table. The events can be filtered based on:

- Level
- Thread name
- Logger
- Message
- NDC

All the details for each event can be displayed by selecting
the event in the table.

Chainsaw also supports loading a events logged to a file using
the XMLLayout format. This
is great for analysing log files, and means you do not need to
keep Chainsaw running continously. It is easy to add support
for loading events from other sources like JDBC.

A picture is worth a thousand words:

.

Finally, why is it called chainsaw?
Because it cuts your log (file) down to size. :-)

## Requirements

Chainsaw is based on the Swing API which requires JDK 1.2 or later.

## Running chainsaw

### Setup

You need to include the `log4j.jar` in the classpath.

### Usage

The command line usage is:

```
  java -D<property>=<value> org.apache.log4j.chainsaw.Main
```

The default behaviour of chainsaw can be changed by setting system properties
using the `-D<property>=<value>` arguments to java. The
following table describes what properties can be set:

|  |  |
| --- | --- |
| **Property** | **Description** |
| chainsaw.port | Indicates which port to listen for connections on. Defaults to "4445". |

## Configuring Log4J

You will need to configure log4j to send logging events to
Chainsaw. Here is a sample `log4j.properties` file
for sending logging events to Chainsaw.

```
log4j.rootLogger=DEBUG, CHAINSAW_CLIENT

log4j.appender.CHAINSAW_CLIENT=org.apache.log4j.net.SocketAppender
log4j.appender.CHAINSAW_CLIENT.RemoteHost=localhost
log4j.appender.CHAINSAW_CLIENT.Port=4445
log4j.appender.CHAINSAW_CLIENT.LocationInfo=true
```
