# Supplementary material for: Visual setup of logical models of signaling and regulatory networks with ProMoT
Source: BMC Bioinformatics. 2006 Nov 17;7:506. doi: 10.1186/1471-2105-7-506 (PMC1665465; doi:10.1186/1471-2105-7-506)
Supplement: Additional File 2 — ProMoT's source. The source code of ProMoT is attached. ProMoT binaries, source, and ProMoT binaries plus all additional libraries (e.g. java) can be downloaded from ProMoT's web page (see Availability and requirements section). [file 1471-2105-7-506-S2.bz2 › Promot/promot/src/gui/org/apache/log4j/config/package.html]

Package used in getting/setting component properties.
