# Supplementary material for: Visual setup of logical models of signaling and regulatory networks with ProMoT
Source: BMC Bioinformatics. 2006 Nov 17;7:506. doi: 10.1186/1471-2105-7-506 (PMC1665465; doi:10.1186/1471-2105-7-506)
Supplement: Additional File 2 — ProMoT's source. The source code of ProMoT is attached. ProMoT binaries, source, and ProMoT binaries plus all additional libraries (e.g. java) can be downloaded from ProMoT's web page (see Availability and requirements section). [file 1471-2105-7-506-S2.bz2 › Promot/promot/src/gui/org/tigris/gef/demo/SpawnedApplet.html]

GEF v0.6 Demo: SpawnedApplet


# GEF v0.6 Demo: SpawnedApplet

Copyright (c) 1996-1998 Regents of the University of California. All
rights reserved.

This software was developed by the Arcadia project at the University
of California, Irvine.

Copyright (c) 1996-99 The Regents of the University of California. All
Rights Reserved. Permission to use, copy, modify, and distribute this
software and its documentation without fee, and without a written
agreement is hereby granted, provided that the above copyright notice
and this paragraph appear in all copies. This software program and
documentation are copyrighted by The Regents of the University of
California. The software program and documentation are supplied "AS
IS", without any accompanying services from The Regents. The Regents
does not warrant that the operation of the program will be
uninterrupted or error-free. The end-user understands that the program
was developed for research purposes and is advised not to rely
exclusively on the program for any reason. IN NO EVENT SHALL THE
UNIVERSITY OF CALIFORNIA BE LIABLE TO ANY PARTY FOR DIRECT, INDIRECT,
SPECIAL, INCIDENTAL, OR CONSEQUENTIAL DAMAGES, INCLUDING LOST PROFITS,
ARISING OUT OF THE USE OF THIS SOFTWARE AND ITS DOCUMENTATION, EVEN IF
THE UNIVERSITY OF CALIFORNIA HAS BEEN ADVISED OF THE POSSIBILITY OF
SUCH DAMAGE. THE UNIVERSITY OF CALIFORNIA SPECIFICALLY DISCLAIMS ANY
WARRANTIES, INCLUDING, BUT NOT LIMITED TO, THE IMPLIED WARRANTIES OF
MERCHANTABILITY AND FITNESS FOR A PARTICULAR PURPOSE. THE SOFTWARE
PROVIDED HEREUNDER IS ON AN "AS IS" BASIS, AND THE UNIVERSITY OF
CALIFORNIA HAS NO OBLIGATIONS TO PROVIDE MAINTENANCE, SUPPORT,
UPDATES, ENHANCEMENTS, OR MODIFICATIONS.

If you have questions or comments, or if you would like to have a copy
of the source code, please return to 
The Graph Editing Framework Homepage.
